# Rosuvastatin-Induced Oral Ulcer: A Case Report and Review of Literature

**DOI:** 10.1155/2022/7960513

**Published:** 2022-03-29

**Authors:** Metab Algeffari, Mansour Alsharidah

**Affiliations:** ^1^Department of Family and Community Medicine, College of Medicine, Qassim University, Saudi Arabia; ^2^Department of Physiology, College of Medicine, Qassim University, Saudi Arabia

## Abstract

**Background:**

The benefits of prescribing statins are well published in the treatment of hypercholesterolemia. With such widespread usage of statins, physicians may be ignoring or misdiagnosing the association of oral side effects with these medications. *Case Summary.* A 54-year-old man presented with a painful ulceration on the dorsum of his tongue that had been recurring for 10 months. Originally, he experienced a burning sensation on his tongue, and as the lesion advanced, the pain became more intense specially when consuming spicy or acidic foods. He is on rosuvastatin for the treatment of hypercholesterolemia for over five years. Several months prior to the lesion forming, his physician increased his daily dosage of rosuvastatin from 10 mg to 20 mg. Four weeks later at a follow-up appointment, all workup did not show any significant findings, the examination revealed a new ulcer on the dorsum of the tongue, and he reported no improvement after antifungal lozenges, nor when administered a short one-week treatment with oral steroids. After eight weeks of statin discontinued, the patient showed improvement with no episodes of ulceration.

**Conclusion:**

Physicians do note that statins affect multiple immunological pathways, which could explain some adverse cutaneous reactions. More research is needed in discovering the link of statins and oral disorders.

## 1. Introduction

The benefits of prescribing statins are well published in the treatment of hypercholesterolemia [1]. The American Heart Association reports that prescriptions for statins have increased during the past 10 years to 64.9 percent, with prescriptions increasing to 221 million from 134 million, especially in elderly patients [2]. Statins work to lower lipids through inhibition of 3-hydroxy-3-methylglutaryl coenzyme-A (HMG-CoA) reductase [3, 4]. While such widespread usage of cholesterol-lowering drugs has been documented as beneficial, physicians may be ignoring or misdiagnosing the association of oral side effects with these medications.

We present in this report a case of a patient with long-term therapeutic use of rosuvastatin who incurred unusual oral ulcers after an increase in the dosage of rosuvastatin.

## 2. Case Presentation

In our clinic, a 54-year-old man recently presented with a painful ulceration on the dorsum of his tongue that had been recurring for 10 months. Originally, he experienced a burning sensation on his tongue, and as the lesion advanced, the pain became more intense, especially when consuming spicy or acidic foods. He reported no improvement after antifungal lozenges were prescribed or when administered a short one-week treatment with oral steroids (20 mg/once per day).

The patient did not have an alcoholism or smoking history. His personal and family history showed no evidence of atopic dermatitis or psoriasis, and no new contact exposures, such as new detergents or other similar substances, had been introduced recently. No new supplements or medications were being taken and thus were ruled out as the cause.

The medications he took daily were as follows: aspirin (81 mg), hydrochlorothiazide (25 mg), rosuvastatin (20 mg), colchicine (0.6 mg), and allopurinol (300 mg). He mentioned that his rosuvastatin therapy for the treatment of hypercholesterolemia had lasted over five years. Several months prior to lesion formation, his physician increased his daily dosage of rosuvastatin from 10 mg to 20 mg.

**Figure 1:**
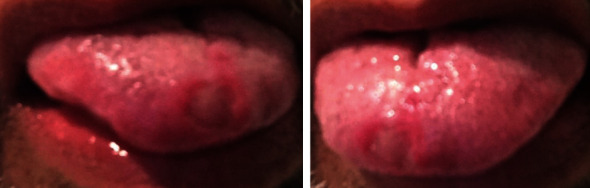
Ulcerative lesion with red halo on the dorsum surface of the right side of the tip of the tongue.

**Figure 2:**
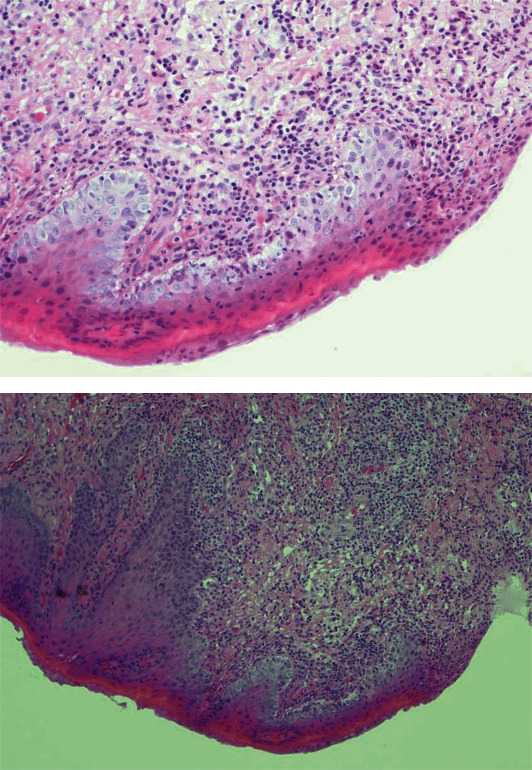
Photomicrograph showed no evidence of squamous cell carcinoma. Bordering the ulcer, granulation tissue presented with a focal collection of inflammatory infiltrates with some atypical cells.

Upon clinical examination, a well-defined 1 cm-by-1.5 cm ulcerated area was evident on the dorsal surface of the right side of the tip of the tongue (Figure 1). The clinical differential diagnosis for the persistent ulcerative lesion included traumatic ulcer, medication-induced ulcer, mucocutaneous ulcerative lesion, chronic ulcerative stomatitis, and oral squamous cell carcinoma. After an evaluation of the patient's nails and skin, no significant findings were found which ruled out mucocutaneous lesions. The patient reported no trauma to the area, and no evidence of sharp tooth or filling was detected upon clinical exam. For histological evaluation, a punch biopsy (5 mm) was performed from the tip of his tongue, which showed no evidence of squamous cell carcinoma. Bordering the ulcer, granulation tissue presented with a focal collection of inflammatory infiltrates with some atypical cells (Figure 2).

Four weeks later, at a follow-up appointment, the patient reported no improvement, and the examination revealed a new aphthous-type ulcer on the dorsum of the tongue. Panel tests ordered for hepatitis markers, an autoimmune panel, prostate-specific antigen, thyroid hormone, complete blood count, and serum protein electrophoresis revealed no significant findings.

To rule out statins as a potential cause for the ulcerations, the patient agreed to discontinue the use of rosuvastatin on a trial basis. After eight weeks, the patient showed improvement with no episodes of ulceration during the trial.

Many patients with oral ulcerations may present with complex polypharmacy; however, statins can generally be discontinued with no immediate complications. Thus, a causative link can potentially be established or rejected.

## 3. Discussion

Although statins have revolutionized the treatment of hypercholesterolemia by inhibiting 3-hydroxy-3-methylglutaryl coenzyme-A reductase, some side effects of oral symptoms have been documented [5–8]. In another study [9], 17 out of 26 hypercholesterolemia patients between the ages of 50 and 70 with oral symptoms reported relief after controlled 7- and 15-day trials of the suspension of statins. Improvement in symptoms was noted as early as three days after statin treatment stopped.

Other oral-related symptoms have occurred in patients taking statins. In one case [10], a 62-year-old man presented with a twelve-month history of a recurrent keratotic lesion with areas of small ulceration on the right lateral border of the tongue. A couple of months prior to the ulcer development, the patient was placed on an atorvastatin regimen due to hypercholesteremia. After panel tests and a punch biopsy, candidiasis with focal ulceration was suggested. Systemic fluconazole and topical nystatin were given but without improvement. When atorvastatin treatment ceased, the patient's symptoms gradually faded over a six-week period.

In another case [11], a 59-year-old female with a history of hypercholesterolemia and hypertension presented with bilateral pruritic, populous eruption on the dorsal aspects of her forearms, wrists, hands, and soles and, to a lesser extent, on her trunk and thighs. She had yellowish-white, lacework-forming streaks on the oral mucosa. No other oral lesion was noted nor other mucosa, nail, or scalp involvement. A topical corticosteroid resulted in temporary improvement. Although the skin and mucosal lesions were characteristic of idiopathic lichen planus, the authors hypothesized that fluvastatin could be a causative factor. Finally, the physicians suspended her fluvastatin treatment, and within 3 weeks, the cutaneous and mucosal lesions resolved slowly. Her cholesterol level rose again, and hypercholesterolemia treatment was changed to lovastatin (20 mg/day), leading to recurrence of a similar lesion that improved within 3 weeks of lovastatin treatment discontinuation.

Physicians should be aware of oral and skin disorders associated with this class of medications. In this particular case study, steroids and antifungal lozenges appeared to have no effect on the treatment of oral lesions. The prevalence of oral symptoms and disorders in patients undergoing statin treatment is presently unknown. Clinicians do note that statins affect multiple immunological pathways, which could explain some adverse cutaneous reactions. Additional research is needed to discover the link between statins and oral disorders.

## Data Availability

The data used to support the findings of this study are available from the corresponding author upon reasonable request.

## Additional Points

*Core Tip*. *Scientific Rationale for the Study*. Many patients with oral ulcerations may present with complex polypharmacy including statin, and the association of oral symptoms and disorders in patients undergoing statin treatment is presently unknown. *Principal Findings*, This is a rare report of a long-term therapeutic case of a patient using rosuvastatin who incurred unusual oral ulcers after an increase in dosage of rosuvastatin. *Practical Implications*. Physicians should be aware of oral and skin disorders associated with this class of medications which can generally be discontinued with no immediate complications. Thus, a causative link can potentially be established or rejected.
